# Sleep deprivation effects on basic cognitive processes: which components of attention, working memory, and executive functions are more susceptible to the lack of sleep?

**DOI:** 10.5935/1984-0063.20200049

**Published:** 2021

**Authors:** Aída García, Jacqueline Del Angel, Jorge Borrani, Candelaria Ramirez, Pablo Valdez

**Affiliations:** Universidad Autónoma de Nuevo León, Laboratory of Psychophysiology, School of Psychology - Monterrey - Nuevo León - Mexico.

**Keywords:** Sleep Deprivation, Cognitive Science, Attention, Memory, Executive Function

## Abstract

**Introduction:**

Sleep deprived people have difficulties to perform daily activities. Their performance depends on three basic cognitive processes: attention, working memory, and executive functions.

**Objectives:**

The aim of this study was to identify which speciﬁc components of these cognitive processes are more susceptible to a 24-h sleep deprivation period.

**Material and Methods:**

Participants were 23 undergraduate students assigned to one of two groups: a control group (n=11, age=18.73±1.62 years) and a sleep deprivation group (n=12, age=18.08±1.16 years). After sleeping freely, control group participants performed a continuous performance task to evaluate the components of attention, a phonological and a visuospatial tasks to record these components of working memory, and a Stroop-like task to assess cognitive inhibition and ﬂexibility, two components of executive functions, at noon for 3 days. Whereas, the sleep deprivation group participants performed the same tasks at noon: after sleeping freely for one night, after a 24-h sleep deprivation, and after one recovery night.

**Results:**

After the sleep deprivation, participants had a signiﬁcant reduction in tonic alertness, selective and sustained attention, components of attention; and in cognitive inhibition, component of executive functions.

**Conclusion:**

A 24-h sleep deprivation period reduces several speciﬁc components of the basic cognitive processes, which are crucial for performing many everyday activities, thus increasing the risk of errors and accidents.

## INTRODUCTION

Sleep is crucial to maintain people’s cognitive performance during wakefulness, when they are carrying out their daily activities, such as studying and working. Adolescents show a phase delay in their sleep-wake cycle during free days (weekends)^1^. In addition, they frequently suffer a reduction of sleep during weekdays because they go to bed late, but they have to wake up early to comply with the school start time^2^. Hence, it is important to study how adolescents’ cognitive performance is affected by the lack of sleep.

Total sleep deprivation for more than 24-h decreases human performance on a variety of tasks and activities, such as: response speed (reaction time), memory, verbal comprehension, as well as the efficiency to perform mathematical operations^3^^-^^7^. Performance on all these tasks and activities may be compromised by the alteration on three basic cognitive processes: attention, working memory, and executive functions^8^^-^^11^. Further, brain damaged patients with a disorder in any of these three basic cognitive processes, show a reduction on the execution of most neuropsychological tasks and tests^12^^,^^13^, that assess more complex processes, such as language comprehension and expression, reading, writing, learning, arithmetic calculations, long term memory, and thought processes.

The discussion of total sleep deprivation effects on performance has been centered on two basic cognitive processes: attention and executive functions. On one hand, studies propose that total sleep deprivation primarily affects attention, while executive functions remain preserved^14^. Therefore, people can respond to demanding situations, but they have trouble responding efficiently to monotonous tasks, due to attentional deficiencies. On the other hand, different studies discuss that sleep deprived people can perform simple tasks, but they have difficulties to accomplish complex tasks, in which executive functions are implicated^15^^,^^16^. Nevertheless, those studies do not consider that each cognitive process has several components. Attention has four components, tonic alertness, phasic alertness, selective attention, and sustained attention^17^. Working memory has two storage components, phonological and visuospatial, a central executive and an episodic component^18^. Executive functions include several components, such as initiative, planning, cognitive inhibition, cognitive flexibility, and self-monitoring^19^^,^^20^. Hence, it is important to study the effects of total sleep deprivation on each component of the basic cognitive processes.

Even though previous studies have found 24-h sleep deprivation effects on these three basic cognitive processes, only few papers analyze the effects on specific components of these cognitive processes, to identify which components are more affected^21^. Additionally, few studies analyze total sleep deprivation effects through a comparison with a matched control group^22^^-^^24^.

It is important to mention that this study analyses several components of these cognitive processes but does not intend to examine exhaustively all the components of these processes. The following sections review the components of the three cognitive processes, attention, working memory and executive functions, as well as the studies that have documented total sleep deprivation effects in these components.

### Attention

Attention is the capacity to process and respond to environmental stimuli and has four components: tonic alertness, phasic alertness, selective attention, and sustained attention^17^^,^^25^. Each component refers to a specific capacity to respond: to any stimuli occurring in the environment (tonic alertness), after a warning signal (phasic alertness), to a specific stimulus (selective attention), and to keep responding efficiently for prolonged periods (sustained attention). Tonic and phasic alertness are related to the arousal system (brainstem, thalamus), while selective attention and sustained attention relate to the prefrontal cortex and parietal cortex^26^^,^^27^.

Total sleep deprivation effects on tonic alertness have been observed through a psychomotor vigilance test, which basically measures reaction time presenting stimuli at the participant’s pace^28^^,^^29^. Total sleep deprivation of 24-h increases reaction time, as well as the frequency of lapses^6^^,^^23^, that are omissions or responses with an excessively longer reaction time^30^. On the other hand, evidence of total sleep deprivation effects on phasic alertness was found only after more than 2 days (54-h) without sleeping^30^, while another study did not find a 24-h sleep deprivation effects on this component of attention^31^. Whereas, a 24-h sleep deprivation effect on selective attention has been observed^32^. Many tasks can be used to assess sustained attention, but specific indices of these cognitive processes must be obtained, such as variability of correct responses, variability of reaction time or changes in performance with time on task^33^.

Total sleep deprivation of 24-h reduces sustained attention as measured by an increase in reaction time with time on task^34^, and more variable reaction times^23^, indices that were obtained through the performance in a psychomotor vigilance test. In a previous study, an efficiency reduction with time on task and an increment in the efficiency variability was observed after 28-h without sleeping^35^. Furthermore, all components of attention showed a reduction during a 30-h recording period in which the participants remained awake^25^^,^^36^. These results suggest that total sleep deprivation affects the four components of attention, but it is still unclear which components of this basic cognitive process are more susceptible to the lack of sleep.

### Working memory

Working memory is the capacity to retain and use information for a brief period. This process also has different components, a phonological storage, a visuospatial storage, an episodic component, and a central executive system^18^. The phonological storage processes verbal information and depends on the left temporal cortex^37^^,^^38^. Visuospatial storage is responsible for retaining visual and spatial information, and it is mainly related to the parieto-occipital cortex^39^. The episodic component participates in the integration and transfer of information between the two storages, while the central executive system selects relevant information and directs it to each memory storage; these components have been related to the prefrontal cortex^18^. It has been observed that total sleep deprivation impairs the central executive system using N-back tasks^40^. Additionally, it has been also found that a 24-h sleep deprivation affects tasks that involve the phonological component^41^, but the results of the sleep-deprived participants were not compared with a control group, while the visuospatial component remained unaffected after 38-h of prolonged wakefulness^42^.

Many authors consider that working memory is required for executive functions, but not all the components of this process are part of the executive functions. Working memory has two storage components: phonological and visuospatial, that are not part of the executive functions. In the present study, the central executive (that is considered a part of executive functions) and the episodic component of working memory were not analyzed. Nevertheless, specific components of executive functions were studied separately.

### Executive functions

Executive functions are the capacity to program, coordinate and supervise our own behavior, according to the environmental requirements. Executive functions have been associated with the prefrontal cortex^43^. These functions also have components such as cognitive inhibition and flexibility, as well as prevision and self-monitoring^19^^,^^44^. Two of these components are crucial to carry out all our activities: cognitive inhibition, the capacity to restrain actions directed to irrelevant goals, and cognitive flexibility, that refers to the capacity to modify the response strategy to cope with the environmental demands^10^. Patients with frontal lobe damage have difficulties in tasks involving cognitive inhibition and flexibility^20^.

Total sleep deprivation effects have been found on decision making tasks, without specifying the components of executive functions involved in resolving them^45^^,^^46^. Other studies have found that a 24-h sleep deprivation reduces the accuracy of switching tasks^47^, but not the switch cost, assessed through reaction time increment^48^. Switching tasks are related to cognitive flexibility, but other processes are also relevant to perform those tasks, such as selective attention^49^. On the other hand, a decrease in motor inhibition^50^^,^^51^, and on cognitive inhibition was observed with time awake for periods longer than 24-h^52^. Nonetheless, sleep deprivation of up to 40-h did not have an effect on several versions of the Stroop task, that is considered to measure cognitive inhibition^22^^,^^53^. Due to the discrepancies in the results observed comparing previous studies, it is important to determine the effects of 24-h total sleep deprivation in these components of executive functions with a computerized version that allow us to determine the effects on precision, as well as on response time.

Another aspect that is important to regard is the fact that many tasks used to assess executive functions cannot be answered more than once because novelty is an essential element for these tests^54^. Hence, performance on these tests cannot be compared on different applications (control, sleep deprivation). These tasks failed to observed sleep deprivation effects mainly because they were designed to observe neuropsychological disorders, and they were not useful to study healthy participants^24^. Therefore, the use of unpredictable randomized tasks is required to compare performance on different conditions.

The present study intends to analyze which components of the basic cognitive processes are more vulnerable to a 24-h sleep deprivation, since previous studies have stated that our brain systems have different degrees of vulnerability to the lack of sleep^55^. It has been proposed that the brain regions that are more susceptible are the frontal and parietal cortex, as well as the arousal system, but the total sleep deprivation effects in the capacity to respond to the tasks associated to those brain areas are inconsistent, some are clearly affected by the lack of sleep, but others remain unaffected^56^. Possibly, the tasks used to assess the cognitive processes are not suitable to evaluate a specific component of each process. Therefore, the use of tasks known to measure specific components of the three basic cognitive processes is relevant to clarify this problem.

In addition, many studies in this field do not include a control group in their protocol, that is, other participants studied in the same conditions and with the same number of applications but without sleep deprivation. The control group is important to separate the sleep deprivation effects from other effects due to repeated applications of the tasks, such as learning, fatigue or boredom. Furthermore, it is important that the performance of the control group and the sleep deprivation group is observed at the same time of the day for the different recording sessions, to observe the effects of sleep deprivation with the less possible influence of circadian rhythms^8^^,^^57^^,^^58^. Total sleep deprivation protocols with prolonged periods (up to 96-h without sleeping) found that performance deteriorated on all activities. Thus, the present study included a control group as well as a 24-h sleep deprivation protocol, without previous sleep reduction, to identify which components of the basic cognitive processes are more vulnerable to the absence of sleep, and which components remain unchanged.

In summary, a sleep deprivation of 24-h or more has been found to affect many tasks related to attention, working memory and executive functions. Nevertheless, it is necessary to study specific components of these processes to analyze the importance of sleep for each of them, analyzing the changes in these components in the same group of people, and comparing these results with a control group, in order to determine which of the components of these cognitive processes are more vulnerable to the lack of sleep. Therefore, the aim of this study was to determine the effects of a 24-h sleep deprivation period on several specific components of attention, working memory and executive functions, and to determine which of these components are more affected by not sleeping.

## MATERIAL AND METHODS

### Participants

In this study, 23 undergraduate students participated voluntarily. They attended school in a variable schedule (07:00-17:00h), from Monday to Friday. The participants were assigned to a control group (n=11, 8 women and 3 men, age=18.73±1.62 years, mean±standard deviation), or to a 24-h sleep deprivation group (n=12, 8 women and 4 men, age=18.08±1.16 years). At the start of the study, participants reported that they did not have any health problems or sleep disorder, and that they did not consume medications or drugs that affect the central nervous system. Each student signed an informed consent letter; the parents of minors also signed this letter. The project was approved by an academic committee at the University and was carried out in accordance with the principles of the Declaration of Helsinki.

### Instruments

The following questionnaires were used: (1) a general information questionnaire, which requested age, school schedule, health condition, alcohol, tobacco, and other drugs consumption; (2) a sleep disorders questionnaire^59^, which consisted in 14 questions. Some of the questions were designed to detect symptoms of insomnia, such as difficulties to fall asleep or to continue sleeping after awakening during the night. Other questions were designed to detect excessive daytime sleepiness, such as sleepiness at waking up, or sleeping too much time. Finally, the other questions were designed to detect some parasomnias, such as snoring, having nightmares, sleep paralysis, sleep talking, or sleepwalking. The participants were required to report the presence of a disorder answering yes or no. For each question that the participants answered affirmatively, they then selected their discomfort degree in a 5-point Likert type scale: none, low, medium, high, or too high discomfort. A symptom was considered as an indicator of a sleep disorder when the person reported the presence of the disorder and a high or too high level of discomfort; (3) a Spanish version of the morningness-eveningness questionnaire^60^^,^^61^, to determine the participants chronotype; (4) a visual analog scale to assess subjective sleepiness^62^, that consisted of a 10cm long horizontal line on which participants mark their current level of sleepiness, the minimum level was represented on the left end and the maximum level on the right end of the line; (5) a sleep diary, to record their bedtime, waking time, and naps daily. Several studies have demonstrated that sleep characteristics obtained with a sleep diary are highly correlated to the results from actigraphy^63^ and polysomnography^64^^,^^65^.

### Tasks

*Continuous performance task (CPT).* The task used in this study was a modified CPT^25^. In this task, single digit numbers were presented randomly in the center of a computer screen for 100ms, while the inter-stimulus interval varied randomly from 1,000 to 1,400ms. Participants were required to press key number one to any number appearing on the screen except 9; to press key two when a 9 appeared; and to press key three when a 4 appeared after a 9. Total task duration was 11.7min. According to definitions stated in the model of attention proposed by Posner and Rafal^17^, responses to any number (other than 9) are indices of tonic alertness, responses to the number 9 are indices of selective attention, and responses to the number 4 after the 9 are indices of phasic alertness. Finally, the standard deviation of correct responses and reaction time throughout the task (stability of responses) is an index of sustained attention^35^. The task had 27 blocks and 20 events per block. On each block the number 9 appeared four times, while the number 4 after a number 9 appeared two times, the remaining fourteen numbers were randomly assorted 0-8 numbers.

*Phonological working memory task*^66^. Each trial of this task started with a visual fixation mark in the center of the computer screen (+), followed by four capital letters presented for 300ms. Then, an interference stimulus appeared for 3,000ms (one-digit number), and finally, a lower-case letter appeared in the center of the screen for 2,000ms. The participants had to respond if the lower-case letter presented at the end was included in the upper-case letters from before. The comparison of a lower-case with the upper-case letters is an important evidence that participants are using a phonological processing to store and retrieve the items of this task. The task consisted of 8 blocks with 8 trials each, 64 trials in total, with a 50% matching rate, and its duration was 6.2min. Trials in each block were randomly presented every time the task was applied.

*Visuospatial working memory task*^66^. In each trial of this task first appeared a visual fixation mark in the center of the computer screen (+), followed by 3 dots in different locations of the screen for 300ms. Then, an interference stimulus appeared for 3,000ms (one-digit number), and finally, a circle appeared in a specific position on the screen for 2,000ms. Participants had to respond if the circle occupied the space of one of the dots previously presented. The task duration was 6.2min, formed by 64 trials, 8 blocks with 8 trials each, with a 50% matching rate. Every time the task was presented, trials in each block were randomly sorted. Both working memory tasks have been used previously to demonstrate the phonological and visuospatial storages, and their relationship with the activation of specific brain regions^67^, as well as to identity age related changes in working memory^68^.

*Computerized Stroop-like task*^52^. In this task, numbers 1 or 2, in blue or red, were displayed at the center of the screen for 100ms, with a random inter-stimulus interval of 1,400-1,600ms. Participants responded by pressing the key number one or the key number two according to the instructions of the 3 following sections: (1) match section. Participants had to press the key with the same number that appeared on the screen. This section was intended to induce a facilitation set; (2) no-match section. Participants had to press the key with the number that is different from the one on the screen, thus inhibiting the tendency to answer with the matching key; (3) shifting section. In this section, the participants had to change the response criteria according to the color of the number on screen. If the number was blue, they had to press the key with the same number that the one appearing on the screen, but if the number was red, they had to respond with the no-matching key. Stimuli were presented in trials that consisted of 3 to 5 consecutive numbers, which were displayed with the same color followed by a different color number (shift stimulus). Trials were randomly sorted within the sections each time the task was presented. The match section consisted of 80 stimuli as well as the no-match section, while the shifting section had 320 stimuli, including 64 shift stimuli. All sections had a 50% matching rate. Correct responses and reaction time of the no-match section were considered indices of cognitive inhibition, while correct responses and reaction time to shift stimuli of the shifting section were considered as indices of cognitive flexibility. The duration of the match and no-match sections was 2.15min each, while the shifting section lasted for 8.5min.

For each one of the tasks used in this study a randomization of the events (stimuli) was carried out. In the CPT the stimuli were randomized for each block, in the phonological and visuospatial working memory tasks the trials were randomized in each block, while in the computerized Stroop-like task the trials were randomized in each section. Consequently, each time the tasks were presented, the order of the stimuli was unpredictable, but with the same number of events to measure each component of the cognitive processes. The randomization allowed the application of equivalent versions of the tasks in each session. The versions applied in the successive days, for both the control and the sleep deprivation group, had the same structure, the same number of stimuli (in a different order) and the same level of difficulty. Further, sorting the events avoided the participant’s automatization of responses to the tasks, as several applications were required.

### Procedure

**Figure 1:**
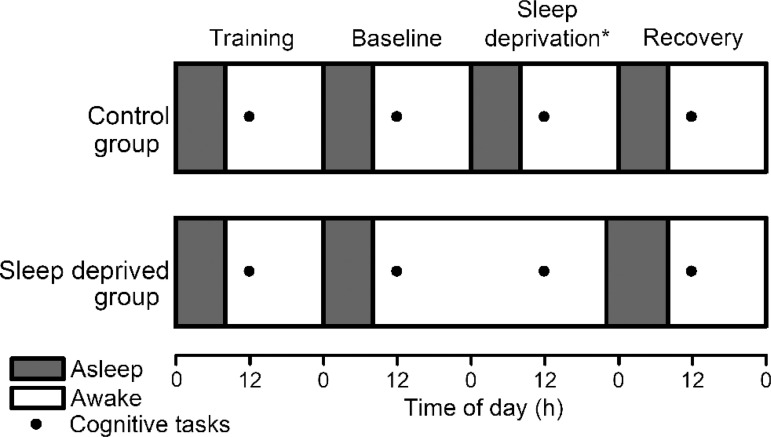
Sleep-wake protocol of the control and sleep deprived group for the training and the three recording sessions. The black dot represents the cognitive tasks and sleepiness scale application. *Control group participants were not sleep deprived.

In a first stage, all participants, and parents of minors signed an informed consent letter, and then, participants answered the general information questionnaire, the sleep disorders questionnaire, and the morningness-eveningness questionnaire. In addition, they kept a sleep diary for 11 consecutive days. In a second stage, participants of both groups were trained individually in the cognitive tasks at the laboratory during the afternoon hours (12:00-17:00h). Afterwards, control group participants answered the sleepiness scale and the cognitive tasks individually in the laboratory at 12:00h (noon) for 3 consecutive days, after sleeping in a self-selected schedule, without sleep restriction. On the other hand, sleep deprivation group responses to the cognitive tasks were recorded individually in the laboratory at 12:00h on the 3 following conditions: (1) baseline, without sleep restriction, they slept in a self-selected schedule and afterwards arrived to the laboratory to respond the tasks at noon; (2) sleep deprivation condition, participants arrived at the laboratory at 20:00h and stayed awake all night, then, they answered the cognitive tasks and the sleepiness scale at 12:00h; (3) recovery condition, participants were recorded again at 12:00h, but after sleeping freely for the night (Figure 1). The participants of the control and sleep deprivation groups responded the tasks in the same order in the three recording sessions: first the participants answered the visual analog scale of sleepiness, then the visuospatial working memory task, then they answered the computerized Stroop-like task, then the phonological working memory task, and finally the continuous performance task. The total duration of each recording session was 45min, for both control and sleep deprivation groups. A control group was included in order to know if the changes observed are due to the sleep deprivation, but not to learning, fatigue, or boredom effects due to the repetition of the same tasks in the different conditions.

Circadian rhythms have been found in most of the components of these three cognitive processes^69^, therefore, this study assessed performance at the same time of day (noon, 12:00h), before and after total sleep deprivation, as well as after one night of sleep recovery for the sleep deprivation group, and also at the same time of day (12:00h) in the three sessions of the control group. All participants answered the cognitive tasks individually sitting in a common office chair in an isolated room, with the 17” screen monitor of the computer on a desk at 60cm in front of them. All tasks were presented and responses were recorded using the Superlab 2.0 software^70^. Room light was ~150 lux and room temperature was 24±1°C.

### Data analysis

A student’s t test was used to analyze the differences between the groups in age and chronotype scores. An analysis of variance (ANOVA) was used to compare the effects of the session factor as repeated measures (baseline, sleep deprivation, and recovery) and the group factor (control, sleep deprived) over the sleep-wake cycle, subjective sleepiness, as well as the components of attention, working memory, and executive functions. Significant interactions between the factors were further analyzed with a post hoc Fisher test. The effect size (partial eta squared, ηp^2^) of the significant comparisons were also obtained to know which components were more affected by the total sleep deprivation. All statistical analyses were performed using the Statistica 10 software^71^.

## RESULTS

There were no significant differences between the groups in age (control group = 18.73±1.62 years, sleep deprived group = 18.08±1.16 years, t = -1.10, NS, no significant) and chronotype (control group = 46.40±4.14 points, sleep deprived group = 46.33±4.70 points, t=-0.03, NS). None of the participants was classified as extreme morning type or extreme evening type.

### Sleep-wake cycle

The multivariate ANOVA showed significant results in the interaction between the group and session factors on bedtime (F=5.61, *p*<0.05, ηp^2^=0.22) and time asleep (F=14.78, *p*<0.01, ηp^2^=0.42), but not on waking time (F=3.81, NS). Results of the post hoc analysis showed a similar bedtime for the three days of recording of the control group (1st day 24.19±1.30, 2nd day 24.11±0.89, 3rd day 24.20±0.92h), and for the baseline of the sleep deprivation group (24.12±0.24h), while this group went to sleep earlier on the recovery night (21.76±3.83h). No differences were found among sleep duration of the 3 days of recording for the control group (1st day 8.43±1.33, 2nd day 8.65±1.20, 3rd day 8.37±1.27h). The night before the baseline recording, the sleep deprivation group slept a similar amount of time (7.79±0.48h) than the control group on the first day. Nonetheless, during the recovery night, sleep deprived participants slept more (11.17±3.29h) than the baseline, and more than the control group on the third day (Table 1).

**Table 1:** Differences between the control and the sleep deprived group on sleep habits, subjective sleepiness, and the components of attention, working memory, and executive functions.

	Group	Baseline	24-h sleep deprivation	Recovery	ANOVA (F) Interaction group x session	Effect size ηp2
**Bedtime**	Control	24.19 ± 1.30	24.11 ± 0.89	24.20 ± 0.92	5.61*	0.22
(Time of day)	Sleep deprived	24.12 ± 0.24	NO SLEEP	21.76 ± 3.83*		
**Waking time**	Control	8.62 ± 1.33	8.76 ± 0.99	8.57 ± 1.09	3.81 ns	0.16
(Time of day)	Sleep deprived	7.91 ± 0.31	NO SLEEP	8.93 ± 1.07		
**Sleep duration**	Control	8.43 ± 1.33	8.65 ± 1.20	8.37 ± 1.27	14.78**	0.42
(h)	Sleep deprived	7.79 ± 0.48	NO SLEEP	11.17 ± 3.29**		
**Subjective Sleepiness**	Control	3.17 ± 2.39	3.01 ± 2.75	2.63 ± 2.31	9.96***	0.32
(VAS, cm)	Sleep deprived	2.85 ± 2.41	6.56 ± 3.03**	2.62 ± 2.31		
**Attention**						
Tonic alertness						
Correct responses (%)	Control	98.82 ± 1.24	99.16 ± 1.01	99.29 ± 0.67	8.77***	0.30
	Sleep deprived	98.65 ± 1.45	95.76 ± 3.23***	99.26 ± 0.64		
Reaction time (ms)	Control	386.90 ± 50.32	385.77 ± 34.34	368.23 ± 43.51	0.78 ns	0.04
	Sleep deprived	372.26 ± 68.79	355.74 ± 53.11	353.48 ± 53.48		
Phasic alertness						
Correct responses (%)	Control	95.29 ± 4.99	93.94 ± 4.23	95.29 ± 4.00	2.85 ns	0.12
	Sleep deprived	90.24 ± 10.96	85.02 ± 14.21	93.43 ± 6.95		
Reaction time (ms)	Control	391.70 ± 59.48	396.45 ± 56.52	372.78 ± 53.56	0.80 ns	0.04
	Sleep deprived	399.32 ± 56.03	395.35 ± 56.87	395.65 ± 54.19		
Selective attention						
Correct responses (%)	Control	86.87 ± 9.73	89.14 ± 8.65	88.89 ± 8.12	13.86***	0.41
	Sleep deprived	82.49 ± 11.99	69.95 ± 19.39**	86.03 ± 9.58		
Reaction time (ms)	Control	484.16 ± 54.55	481.33 ± 54.27	467.89 ± 59.41	1.51 ns	0.07
	Sleep deprived	474.43 ± 46.79	490.17 ± 35.92	456.84 ±43.35		
Sustained attention (SD)						
Correct responses (%)	Control	0.86 ±0.46	0.82 ± 0.45	0.77 ± 0.31	8.72***	0.30
	Sleep deprived	1.05 ± 0.53	1.61 ± 0.78**	0.94 ±0.40		
Reaction time (ms)	Control	34.20 ± 8.41	34.04 ± 5.27	28.73 ± 5.24	0.03 ns	0.001
	Sleep deprived	38.87 ± 12.76	39.35 ±11.48	33.16 ± 11.73		
**Working memory**						
Phonological storage						
Correct responses (%)	Control	92.36± 8.27	91.10 ± 4.68	95.07 ± 5.68	2.50 ns	0.18
	Sleep deprived	90.29 ± 8.98	79.94 ± 14.35	88.89±7.14		
Reaction time (ms)	Control	840.54 ± 64.84	839.96 ± 93.83	789.52 ± 73.43	0.77 ns	0.01
	Sleep deprived	859.93 ± 159.04	853.35 ± 156.51	829.22 ± 145.91		
Visuospatial storage						
Correct responses (%)	Control	85.38 ± 8.44	83.59 ± 8.55	81.91 ± 7.31	1.22 ns	0.10
	Sleep deprived	84.18 ± 10.46	80.05 ± 7.07	83.80 ± 10.62		
Reaction time (ms)	Control	877.50 ± 145.73	819.49 ±140.68	807.37 ± 115.85	0.07 ns	0.02
	Sleep deprived	889.52 ± 155.45	833.93 ± 133.51	831.57 ± 124.65		
**Executive functions**						
Cognitive inhibition						
Correct responses (%)	Control	94.86 ± 3.05	94.74 ± 3.17	93.38 ±4.29	7.63**	0.29
	Sleep deprived	93.83 ± 3.97	87.48 ± 6.56**	93.85 ± 2.12		
Reaction time (ms)	Control	433.85 ± 58.97	435.31 ± 46.06	422.48 ±52.64	0.05 ns	0.002
	Sleep deprived	445.39 ± 119.0	446.58 ± 70.9	441.78 ± 50.70		
Cognitive flexibility						
Correct responses (%)	Control	86.25 ± 7.06	85.68 ± 9.54	86.99 ± 9.29	1.90 ns	0.09
	Sleep deprived	82.48 ± 8.92	74.59 ± 14.63	83.48 ± 8.28		
Reaction time (ms)	Control	657.46 ± 77.26	644.88 ± 66.89	626.56 ±77.51	0.66 ns	0.03
	Sleep deprived	652.26 ± 101.80	665.41 ±82.40	632.40 ± 96.4		

Values are mean ± standard deviation. ANOVA = Analysis of Variance. VAS = Visual Analog Scale. ηp^2^ = Partial eta squared. ns = No significant differences. Bold values are different from all others on the post hoc analysis for at least:

* p<0.05,

** p<0.01,

*** p<0.001.

### Subjective sleepiness

A significant ANOVA interaction was found between session and group factors on sleepiness (F=9.96, *p*<0.001, ηp2=0.32). According to the post hoc analysis, participants of the sleep deprivation group reported a higher level of sleepiness after being sleep deprived (6.56±3.03cm), in comparison with their baseline (2.85±2.41cm), and after recovery (2.62±2.31cm), as well as it was higher than the 3 reports of the control group (1st day 3.17±2.39, 2nd day 3.01±2.75, 3rd day 2.63±2.31cm). There were no differences on sleepiness among the three days of the control group (Table 1).

### Components of attention

**Figure 2:**
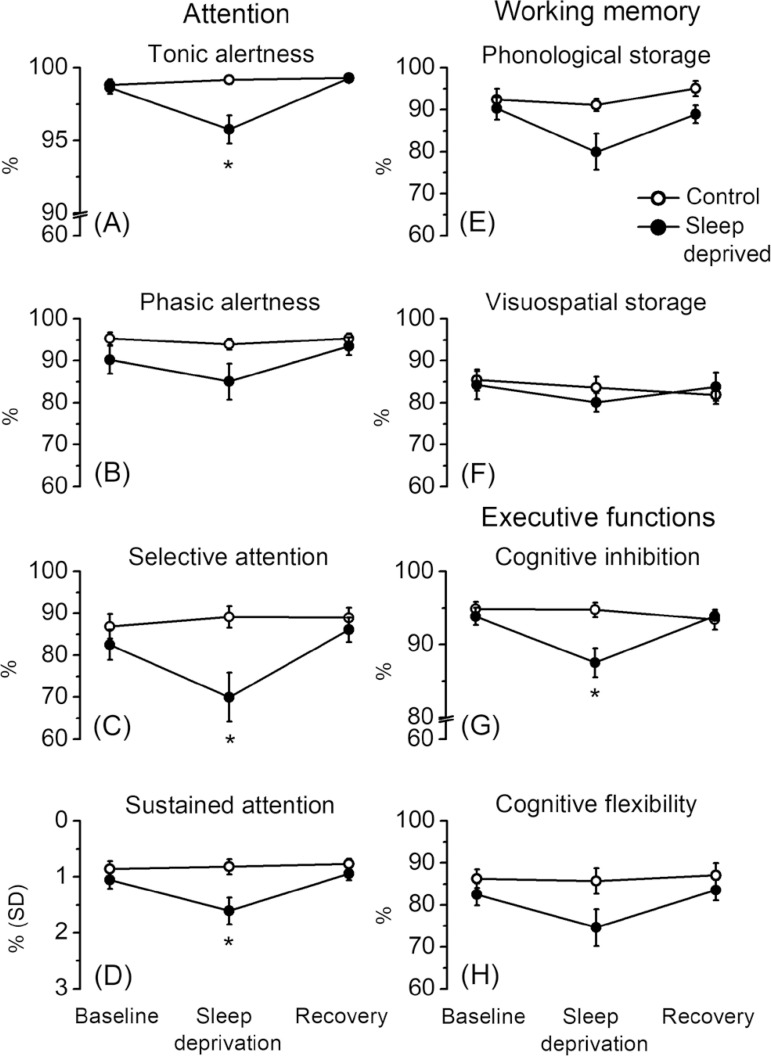
Sleep deprivation effects on the components of attention, working memory and executive functions. White circles represent the control group and black circles represent the sleep-deprived group on the three conditions. Control group participants were not sleep deprived. Values are mean ± standard error of the mean, *p<0.01.

Multivariate ANOVA significant interactions between session and group factors were found for correct responses of tonic alertness (F=8.77, *p*<0.001, ηp2=0.30), selective attention (F=13.86, *p*<0.001, ηp2=0.41), and sustained attention (F=8.72, *p*<0.001, ηp2=0.30). Post hoc analysis showed that after being sleep deprived, participants significantly reduced their correct responses of tonic alertness, from their baseline, and they increased them again in the recovery session (baseline 98.65±1.45, sleep deprivation 95.77±3.23, recovery 99.26±0.64%) (Figure 2A). Their performance after sleep deprivation also was lower than the 3 recordings of the control group (1st day 98.82±1.24, 2nd day 99.16±1.01, 3rd day 99.29±0.67%), which did not have significant differences among them (Figure 2A). Similar results were found on selective attention, with a decrease in correct responses after sleep deprivation (baseline 82.49±11.99, sleep deprivation 69.95±19.39, recovery 86.03±9.58%), and significantly less correct responses than the control group applications (1st day 86.87±9.73, 2nd day 89.14±8.65, 3rd day 88.89±8.12%) (Figure 2C). Sustained attention changes were also observed as a reduction in correct response stability after sleep deprivation, which further incremented on the recovery session (baseline 1.05±0.53, sleep deprivation 1.61±0.78, recovery 0.94±0.40%). Differences were also found between the sleep deprivation session and all the recording sessions of the control group (1st day 0.86±0.46, 2nd day 0.82±0.45, 3rd day 0.77±0.31%), which did not differ among them (Table 1, Figure 2D). On the other hand, correct responses of phasic alertness had no significant interactions between session and group factors (F=2.85, NS) (Figure 2B).

Furthermore, reaction time of all components of attention did not have a significant interaction between session and group factors (Table 1). Nevertheless, a main effect of the session factor was found on tonic alertness (F=3.52, *p*<0.05), selective attention (F=7.10, *p*<0.01), and sustained attention (F=5.38, *p*<0.01). Participants of both groups responded faster and with more stable reaction times as the sessions advanced (Table 1).

### Working memory: phonological and visuospatial storages

Correct responses of the phonological (F=2.50, NS) and the visuospatial (F=1.22, NS) storage components of working memory did not show a significant session and group interaction (Table 1, Figures 2E and 2F). On the other hand, there was a significant main effect among sessions on the reaction time of the visuospatial storage (F=7.70, *p*<0.01). Participants of both groups diminished their reaction time from the first session (883.22±146.73ms) to the second (826.37±134.06ms) and third sessions (818.90±117.71ms).

### Executive functions: cognitive inhibition and flexibility

An ANOVA significant interaction between group and session factors was observed on correct responses of cognitive inhibition (F=7.63, *p*<0.01), but not on cognitive flexibility (F=1.90, NS) (Figure 2H). On the post hoc analysis, a decrease in cognitive inhibition was found after 24-h of sleep deprivation and an increase after one night of recovery (baseline 93.83±3.97, sleep deprivation 87.48±6.56, recovery 93.85±2.12%). Differences were also found between the efficiency of this component of the sleep deprived participants and the three applications of the control group (1st day 94.86±3.05, 2nd day 94.74±3.17, 3rd day 93.38±4.29%), which had no significant differences among them (Figure 2G). On the other hand, a main effect of the session factor was found on the reaction time of cognitive flexibility (F=3.42, *p*<0.05). Participants of both groups responded faster on the third session than on the previous two (Table 1).

Regarding which component of these cognitive processes were more affected by sleep deprivation, the effect sizes results showed that: 24-h sleep deprivation had the largest effect size on selective attention (ηp2=0.41), while a medium effect size was found on tonic alertness (ηp2=0.30) and sustained attention (ηp2=0.30) (components of attention), and on cognitive inhibition (ηp^2^=0.29) (a component of executive functions). No effects of the 24-h sleep deprivation were found on phasic alertness (component of attention), visuospatial and phonological storages (components of working memory), or cognitive flexibility (component of executive functions). Moreover, one night of recovery was enough to counteract the effects of the 24-h sleep deprivation on all these components of the basic cognitive processes.

Apart from group comparisons, sleep deprivation showed a distinct pattern of effect in each individual. After sleep deprivation, three participants presented less correct responses in all components of the three cognitive processes. Four participants presented a reduction in most of the components of the three cognitive processes; two of them did not decrease their visuospatial working memory while the other two participants did not change their phasic alertness. One participant had a decrement in the components of attention (except for phasic alertness) and executive functions. Another participant showed a decrease in working memory, executive functions, and selective attention, while one participant showed a decrease in the components of attention, except for tonic alertness.

## DISCUSSION

On the question of what cognitive processes are primarily affected by total sleep deprivation, results in this study demonstrated that several specific components of two basic cognitive processes, attention and executive functions, are susceptible to the absence of sleep for one night, but with different degrees.

Selective attention, that allow us to respond to a specific stimulus and ignore others, had the largest effect size of all. This component is related to the dorsolateral prefrontal and parietal areas of the brain^27^. Furthermore, sustained attention, the capacity to maintain the performance efficiency through time, was also affected by the 24-h sleep deprivation and is also related to the prefrontal cortex, specifically the right dorsal and medial frontal cortex, as well as the inferior parietal cortex^72^^,^^73^. In addition, cognitive inhibition, which is the capacity to restrain inadequate responses, a component of executive functions, also diminished after the sleep deprivation and is related with the ventro-medial area^74^, the right dorsolateral area^75^, and the right posterior-inferior gyrus of the prefrontal cortex^76^. These components decreased after 24-h of sleep deprivation with a medium effect size. These results confirm the findings of other studies as the prefrontal cortex is a region of the brain vulnerable to the lack of sleep^54^^,^^77^. A component of executive functions, cognitive flexibility, which is also related to the prefrontal cortex, was not affected by the total sleep deprivation. Similar results were found in previous studies, where switch cost was not affected after a total sleep deprivation^48^, known index of cognitive flexibility. The analysis of the components in the present study allow us to determine which components are more susceptible to the lack of sleep and which are more resilient. In this case, the capacity to actively detect a specific stimulus and respond to it for a prolonged period, as well as the capacity to detain pre-learned responses are more affected by the total sleep deprivation than the capacity to actively adjust the responses according to changes in the environment that is preserved. On the other hand, tonic alertness is the capacity to respond to any stimulus and it is associated with the subcortical arousal system, which activates all areas of the brain. This component was also affected by the total sleep deprivation with a medium effect size. This result is similar to the results found in other studies, which confirm that our arousal system, the thalamus and its connections to the brain cortex, is vulnerable to sleep deprivation^32^^,^^78^^,^^79^. Nevertheless, phasic alertness, which is also related to the arousal system, was not affected by 24-h of sleep deprivation. These results are in contradiction with previous studies that have found that a total sleep deprivation affected phasic alertness, but those studies did not compare the results with a control group^80^, while other study found effects only after 54-h without sleeping^30^. On the other hand, a previous study also did not find a 24-h sleep deprivation effect on phasic alertness^31^, as phasic alertness was considered a top-down function from the brain cortex modulating the arousal system.

The results of the present study are in accordance with the brain regions that have been considered as more susceptible to the lack of sleep, frontal and parietal cortex, as well as the arousal subcortical system^56^^,^^79^.

The performance decrease of specific components observed in this study could explain the 24-h sleep deprivation effects observed on many tasks. Thus, a reduced level of efficacy on these components produces limitations to process information from the environment, to perform daily activities, as well as to make decisions.

Total sleep deprivation effects in working memory observed in previous studies^40^^,^^41^, could be due to changes in the prefrontal cortex, therefore the central executive component of working memory is altered, similar than other components that rely on the function of this brain region, such as selective attention and cognitive inhibition observed in this study^81^^,^^82^. Nevertheless, a 24-h sleep deprivation has no effects on the working memory storages, related to posterior brain regions.

According to the results of this study, total sleep deprivation increases subjective sleepiness. This sensation decreases after sleeping freely during the recovery night, in which sleep duration go up for approximately 3h. Similar results have been obtained in practically every study made on this subject^83^^-^^85^. Hence, people are capable to detect and report their sleep propensity, but also, this report could be related to their identification of the reduction in their capacity to respond to the environment. Further studies are needed to analyze the relation of the sleepiness report with the decrement of specific processes and capacities.

The significant effects of total sleep deprivation in the components of cognitive processes were observed on accuracy (correct responses), but not on reaction time. These results confirm previous observations that the total sleep deprivation have a greater effect on accuracy than on speed^86^. On the other hand, a reaction time reduction in components of attention, working memory, and executive functions was observed throughout the sessions, not as a result of the total sleep deprivation, but as a practice effect. That is, participants of both groups responded faster with the practice on these tasks. These results should be considered when reaction time tasks are used to assess cognitive processes, especially in studies that have no control group.

Previous studies have shown that adolescents go to sleep later (phase delay) and sleep less because they have to wake early to go to school^2^. Due to these characteristics, adolescents are more likely to reduce their sleep or even to spend the whole night awake; therefore, the results of this study are particularly relevant for this age group. The results of the present study could explain the lower school grades found in students with greater sleep deprivation^87^.

In studies that analyze people performance on several tasks, the order of application has been taken into consideration because it could change the person’s performance on each of the tasks. Nevertheless, in this study the order of application was not considered as a factor, since it was the same for the three recording sessions, for the control group and the sleep deprivation group (baseline, after total sleep deprivation, and after a sleep recovery night). It is interesting to notice that the task answered at the beginning and less subject to fatigue (visuospatial working memory task) had a lower efficiency than the task applied at the end of the sessions (continuous performance task). This indicates that the order of the tasks did not affect the participants’ performance on them.

Total sleep deprivation could have different effects in accordance with the person’s chronotype^88^. Since the participants of the present study did not have extreme chronotypes, this factor was considered to have no effect on the results.

A limitation of this study was that the effects of the total sleep deprivation were documented in only some components of the basic cognitive processes, further studies are required to analyze other components of these processes. Another issue that is important to study in the future is how the components of these basic cognitive processes are affected by the reduction of sleep for one or several consecutive days. These studies are important because many people around the world live with chronic sleep reduction, in order to comply with school or work schedules, as well as with family and social activities. It has been found that sleep restriction for several days produces a reduction in tonic alertness^6^, and the phonological storage of working memory^89^. Future studies are needed to analyze sleep reduction on other basic cognitive processes and their components.

The objective of this study was to document which specific components are more affected with total sleep deprivation, therefore, the analysis of several components in a sleep deprivation group compared to a control group allowed us to accomplish that goal. Despite the small number of participants, performance of both groups was the same in the three conditions in the components that did not showed changes with the 24-h sleep deprivation, while the effects observed in the components affected are in fact due to the sleep deprivation. Even though previous studies have found total sleep deprivation effects in some of the components analyzed in this study, it is important to take into account that most of those studies did not compare the performance of the sleep deprived participants with a control group, or compared those groups but without a baseline. On the other hand, other studies found effects of total sleep deprivation in phasic alertness, the storages of working memory and the cognitive flexibility only after periods of sleep deprivation longer than 24-h (effects were observed after periods of 48-h or longer)^30^^,^^42^.

The implications of the results of this study are relevant for people that remain awake for more than 24-h. Previous studies have documented that college students report staying awake during the whole night, a practice known as “an all-nighter”^90^. Likewise, truck drivers have also reported remaining awake for more than 20-h, especially on long routes^91^. Even though regulations have been set in some countries, drivers could be on duty for as long as 15 hours^92^.

In these situations, people could have difficulties to respond to the environment in general, due to their tonic alertness diminishment, but especially when they have to respond to specific stimuli, as a result of the decrease on selective attention, and to respond for long periods, because their sustained attention is lower. Furthermore, people could have difficulties when they must restrain inadequate but habitual responses, due to the decrement in cognitive inhibition. Accurate performance for prolonged periods and behavior regulation rely on the components affected by the 24-h sleep deprivation, so they are critical when people perform problem solving tasks and high-risk activities, such as driving vehicles or traffic control, hence the consequences of failing would be serious or even fatal.

In conclusion, total 24-h sleep deprivation reduces tonic alertness, selective attention and sustained attention, components of attention, as well as cognitive inhibition, a component of executive functions. The brain areas that seem more susceptible to total sleep deprivation, related to the affected components, are the prefrontal and parietal cortex, as well as the arousal system of the brain subcortex.
